# Correction to “Harnessing the dual nature of *W. coagulans* for sustainable production of biomaterials and development of functional food”

**DOI:** 10.1111/1751-7915.14475

**Published:** 2024-05-11

Maresca, E., Aulitto, M. & Contursi, P. (2024) Harnessing the dual nature of *W. coagulans* for sustainable production of biomaterials and development of functional food. *Microbial Biotechnology*, 17, e14449. Available from: https://doi.org/10.1111/1751‐7915.14449

The title should be replaced with: “Harnessing the dual nature of *Bacillus* (*Weizmannia*) *coagulans* for sustainable production of biomaterials and development of functional food”, as it was originally provided on the Wiley submission website.

We apologize for this error.

